# Comparison of Diphtheria Cases in Children Before and During the Pandemic Era in Surabaya, Indonesia: A Study of Six-Year Data

**DOI:** 10.7759/cureus.66949

**Published:** 2024-08-15

**Authors:** Dominicus Husada, Kalista W Nuringhati, Sandy G Tindage, Rahma I Mustikasari, Leny Kartina, Dwiyanti Puspitasari, Parwati S Basuki, Ismoedijanto Moedjito, Rosita D Yuliandari, Nanik Sukristina

**Affiliations:** 1 Department of Child Health, Faculty of Medicine Universitas Airlangga, Surabaya, IDN; 2 Department of Child Health, Dr. Soetomo General Academic Hospital, Surabaya, IDN; 3 Department of Infectious Diseases Control, Surabaya Health Office, Surabaya, IDN

**Keywords:** surveillance, vaccine preventable diseases, children, covid-19 pandemic, surabaya, diphtheria

## Abstract

Introduction

Indonesia has a high incidence of diphtheria, especially in children. Surabaya has become a government regional reference center, as it is the capital of East Java province, which has the highest rate of diphtheria across the 38 regions. The aim of this study is to report our six-year pediatric diphtheria data, focusing on comparisons between before and during the pandemic era.

Method

This surveillance report was collected from community health centers and hospitals throughout Surabaya from January 1, 2017 to December 31, 2022. Collected data included demographic characteristics, clinical and laboratory aspects, the health centers, immunization history, and management. As per Indonesian guidelines, the diagnosis of diphtheria in this country requires a positive microbiological culture or approval from the National Experts on Diphtheria Committee.

Results

In total, there were 112 cases, of which 89 were found before the pandemic era. Although the number of cases declined during 2020-2022, the predominant age group, the immunization status, and the most common type of diphtheria remained consistent with pre-pandemic trends. Most cases had incomplete immunization or unimmunized children (67.8%), with the age group of 5-12 years old (44.6%), and with tonsillar diphtheria (83%). The case fatality ratio was 1.8%. Regarding the biovar of *Corynebacterium diphtheriae*, gravis is the most frequent finding.

Conclusion

The incidence of diphtheria cases in children in Surabaya was significantly lower during the pandemic. Although immunization coverage was not better, preventive measures during the pandemic may have played a role. Most patients did not have complete immunization histories during the study period, and the predominant type was tonsillar diphtheria. Since the trend in 2021-2022 increased, routine surveillance is essential.

## Introduction

The COVID-19 pandemic has led to the widespread adoption of preventive measures such as handwashing, mask-wearing, and social distancing [1]. These actions have not only curbed the spread of COVID-19 but have also impacted the incidence of other diseases, especially those transmitted via droplets or airborne particles. In numerous developed nations, the rates of influenza and RSV have plummeted to record lows, while in many developing countries, the cases of measles and diphtheria have significantly declined [2-4].

Indonesia has been grappling with a diphtheria outbreak since 2011, with East Java being one of the most affected provinces, particularly in its northern and eastern regions [5,6]. Surabaya, the capital city located in the northern part of East Java, had a high number of diphtheria cases before the pandemic. Surrounding districts also reported elevated diphtheria cases [5]. The first COVID-19 cases in Indonesia were detected on March 2, 2020, and a national disaster status was declared on April 13, 2020 [7]. Since then, universal preventive measures have become routine.

Diphtheria is a severe vaccine-preventable disease caused by toxigenic strains of *Corynebacterium diphtheriae*, *Corynebacterium ulcerans*, and *Corynebacterium *​​​​​​​*pseudotuberculosis* [8-10]. Before the advent of vaccines, diphtheria was among the deadliest diseases globally. Today, most diphtheria cases are found in the WHO Southeast Asian Regional Office (SEARO) and Africa Regional Office (AFRO) regions [11,12].

Surabaya, with a population of four million, is a crucial area as the provincial capital. The city's diphtheria cases are of significant importance and serve as a key reference for the regional government. This study aims to present and analyze six years of diphtheria data in Surabaya, with a specific focus on comparing the pre- and during-pandemic periods. The findings will be essential for understanding the diphtheria situation in Surabaya and its regional implications.

## Materials and methods

Study design and data source

This cross-sectional study is based on surveillance reports collected from the Surabaya City Health Office and the East Java Provincial Health Office between 2017 and 2022. Reports were obtained from all community health centers, hospitals, and additional sources on a daily, weekly, or monthly basis, depending on the presence of cases. Surabaya has 63 community health centers and fewer than 100 hospitals of various levels; however, only two hospitals, Dr. Soetomo General Academic Hospital and Dr. Ramelan Navy Hospital, accept inpatient diphtheria cases. Suspected diphtheria cases, both outpatient and inpatient, were required to be reported to the health office for follow-up.

Data variable

The collected data included patient identity, demographic characteristics, clinical signs and symptoms, health center facilities involved, previous medication, and immunization history.

Study subjects

We included children and adolescents under 18 years old who resided in the Surabaya region, were diagnosed as diphtheria patients, and were registered at the Surabaya Health Office. According to the Indonesian Ministry of Health, diphtheria diagnosis was based on clinical appearances, which the National Expert Committee confirmed, or microbiological culture [13]. We exclude patients who live and are transferred from other districts outside Surabaya. Clinically, diphtheria presents with a brownish-white pseudomembrane, primarily in the tonsil and pharyngeal area, which is difficult to detach without bleeding. Patients typically exhibit mild fever, sore throat, and other localized symptoms depending on the pseudomembrane's location [8-10,13].

Case definition

According to the current policy of the Indonesian Ministry of Health, a positive case of diphtheria in children requires a positive microbiological culture from a nasal or throat swab (or a swab from the lesion) or approval from the National Diphtheria Expert Committee. Clinical diagnoses had to be approved by this committee, requiring clinicians to send patient data and visual evidence of the pseudomembrane.

Laboratory

Microbiological cultures were conducted at Surabaya's Public Health Laboratory (BBLKM), supervised by the WHO SEARO regional diphtheria laboratory, following WHO Laboratory Guidelines [14]. Specimens were placed in Amies media and sent to the laboratory, where positive cultures were identified by the biotype of *C. diphtheriae* and the toxigenicity test using Modified Elek. During this study period, the laboratory did not test for *C. ulcerans* and *C. pseudotuberculosis*.

Treatment

As per Indonesian guidelines, identified diphtheria cases required at least 10 days of hospitalization, which involved the use of antibiotics, including procaine penicillin and erythromycin, diphtheria antitoxin, isolation procedures, and contact tracing [13].

Statistical analysis

All data were compiled using an MS Excel file, and statistical analyses were performed using IBM SPSS Statistics for Windows, Version 24 (Released 2016; IBM Corp., Armonk, New York, United States). Categorical variables are presented as numbers and percentages of patients, with case fatality rates (CFR) also calculated. Bar diagrams illustrate the distribution of cases over the study period. A p-value less than 0.05 was considered significant.

Ethical approval and consent

This study received ethical approval from the Dr. Soetomo General Hospital Health Research Ethical Committee. Informed consent was waived as no individual data were disclosed.

## Results

**Figure 1:**
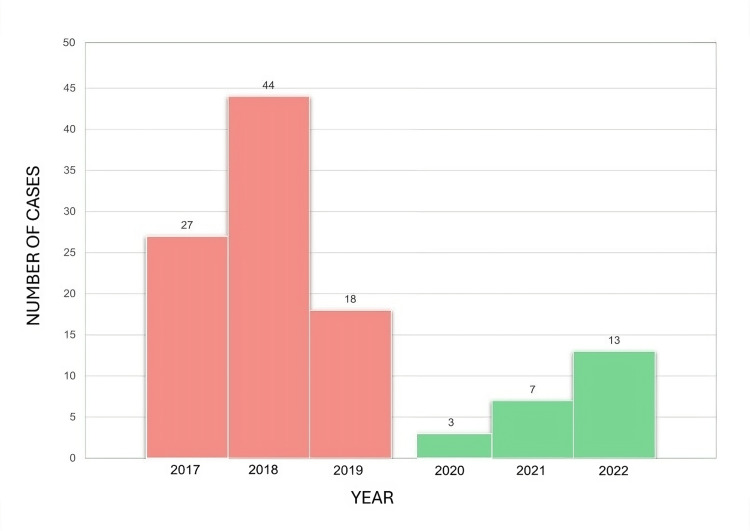
The diphtheria cases in children in Surabaya distribution by year p-value < 0.001 between both groups

There were 112 cases in children from January 1, 2017 to December 31, 2022. Because the culture results were negative and the expert committee did not accept them as diphtheria cases, 23 other patients were excluded. The total number and epidemiological details are revealed in Table 1 and Figure 1.

**Table 1:** Comparison of demographic and clinical data of diphtheria cases in children pre- and during the pandemic period ^*^: p-value of the comparison of two groups, before and during the pandemic; Mann-Whitney U test; ^a^: percentage of the total amount (six years); ^b^: mean, range, and median of age are expressed in months; ^c^: percentage of all age groups in the same period; ^d^: percentage of all sex categories in the same period; ^e^: percentage of all immunization status in the same period; ^f^: percentage of all membrane locations in the same period; ^g^: percentage of all outcomes in the same period n: number of cases; SD: standard deviation

	January 1, 2017 until December 31, 2019	January 1, 2020 until December 31, 2022	Total	p-value^*^
Total number of cases (n (%)^a^)	89 (79.46)	23	112	<0.001
Age^b^ (months):				0.541
Mean + SD	80.67 + 45.67	73.52 + 45	79.2 + 45.42	
Range	12-204	11-168	11-204	
Median	72	60	72	
Age category: (n (%)^c^)				0.240
Less than 12 months	0 (0)	1 (4.4)	1 (0.9)	
12-36 months	18 (20.2)	6 (26.1)	24 (21.5)	
>36-60 months	23 (25.8)	6 (26.1)	29 (25.9)	
>60-144 months	41 (46.1)	9 (39.0)	50 (44.6)	
>144-<196 months	7 (7.9)	1 (4.4)	8 (7.1)	
Sex: (n (%)^d^)				0.092
Boys	63 (70.8)	13 (56.5)	76 (67.9)	
Girls	26 (29.2)	10 (43.5)	36 (32.1)	
Immunization: (n (%)^e^)				0.305
Unimmunized	13 (14.6)	2 (8.7)	15 (13.4)	
Not completed	49 (55.1)	12 (52.2)	61 (54.4)	
Completed by age	26 (29.2)	8 (34.7)	34 (30.4)	
No data	1 (1.1)	1 (4.4)	2 (1.8)	
Location of the membrane: (n (%)^f^)				0.092
Tonsillar	71 (79.8)	22 (95.6)	93 (83.0)	
Tonsil-pharyngeal	0	0	0	
Pharyngeal	5 (5.6)	0	5 (4.1)	
Laryngeal	1 (1.1)	0	1 (0.9)	
Nasal	0	0	0	
Skin	0	0	0	
Others	0	0	0	
No data	12 (13.5)	1 (4.4)	12 (12.0)	
Outcome: (n (%)^g^)				0.3
Survive	88 (8.9)	22 (95.6)	120 (98.2)	
Died	1 (1.1)	1 (4.4)	2 (1.8)	

Two patients died (case fatality rate: 1.8% or 2/112), one from each period. The first child died because of respiratory failure, and the second because of myocarditis. Only four positive microbiological cultures were found among all patients (positivity rate: 3.6% or 4/112). Three cases were biotype gravis, and one was mitis.

## Discussion

Indonesia has experienced multiple outbreaks of vaccine-preventable diseases, including diphtheria, with a significant rise in cases recognized since 2011. East Java stands out as the province with the highest total number of diphtheria cases, with the majority of these cases concentrated in the northern and eastern regions. Surabaya, the capital city of East Java, has also seen a high incidence of diphtheria cases [15,16].

During the COVID-19 pandemic, the incidence of diphtheria among children in Surabaya was markedly reduced, with only about 25% of the cases reported in the three years prior to the pandemic. Despite this decrease, the distribution of age groups, the location of the pseudomembrane, and the immunization status of the affected children remained consistent with pre-pandemic trends.

At least four factors may play a role in disease reduction. First and mostly, the social distancing measures effectively reduce the transmission of the disease. Droplets transmit *C. diphtheriae*; the most contagious area is just 1-3 meters away. The reduction of disease incidence was also seen in many infectious diseases worldwide. Second, the attention of the health officer anywhere in Surabaya, just like in other parts of the world, was for COVID-19 and other infectious diseases may not be prioritized. The impact was a reduction in reporting. Some of the diphtheria cases were underdiagnosed. Mild diphtheria may be cured even spontaneously after several days. Third, many health and medical centers were closed during the pandemic, so disease identification was lacking. Laymen may not easily recognize diphtheria. In this context, there were two possibilities. The report might have decreased significantly despite the present diphtheria, but it was also possible that the disease decreased because of preventive measures during the pandemic. Fourth, people were afraid to go to the doctors or hospitals, so they usually tried to cure themselves with self-medications without seeing any medical officers.

The year 2020 saw the lowest number of diphtheria cases during the pandemic, coinciding with the Indonesian Government's implementation of stringent COVID-19 preventive measures. However, in the subsequent two years, there was a relaxation of these measures, with some individuals even refusing to wear masks.

The predominant age group affected by diphtheria was children aged 5-12 years, reflecting a shift toward older age groups over time, which was also found in many other diphtheria outbreaks worldwide [17-20]. This trend may be attributed to improved immunization coverage and better immunity among infants and young children in Surabaya. According to the Indonesian Ministry of Health's immunization schedule, diphtheria vaccination is administered at least seven times, including three doses during the first year, a booster at 18 months, and three additional doses during elementary school. Despite these efforts, some children began elementary school with incomplete immunization histories [16].

The preventive measures during the pandemic were also effective in reducing the incidence of other respiratory-borne diseases. In developed countries like the USA and the UK, new cases of influenza and RSV reached record lows [2,3,21], a phenomenon also observed in diphtheria cases in this study. Although the total number of cases was low, transmission of the disease could not be entirely halted. This ongoing transmission is likely due to low vaccination coverage and suboptimal immunity among certain populations. As anticipated, a significant portion of the diphtheria cases were in unimmunized or incompletely immunized individuals, a finding consistent with global trends [22-24]. While direct contact with the bacteria can confer some immunity, vaccination remains the most effective method for generating antibodies against diphtheria toxin. In East Java, initial vaccination coverage is generally adequate, but challenges persist with the administration of booster doses. Outbreak response immunizations typically address the missing fourth dose of diphtheria toxoid [16]. There were at least four possibilities regarding diphtheria cases in completely immunized children: the effectiveness of the diphtheria toxoid, which is not 100%, waning immunity, improper vaccine handling, and inaccuracy in the reports since most cases could not show valid evidence of immunization.

Regarding the pseudomembrane’s location, most cases involved the tonsils and pharynx, which also aligns with global patterns [8-10]. Fewer cases were observed in the skin, nasal passages, or other atypical sites. The positivity rate for microbiological cultures was low, with the predominant biovar being *C. diphtheriae *gravis. Although *C. diphtheriae* gravis is known for potential resistance issues [25], such problems were not documented in the hospitalization records during this study period.

There are some implications of these findings. The isolation or social distancing measures are effective in preventing the spreading of diphtheria cases, so these prevention efforts should be prioritized in every new case. However, Surabaya is a dense, populated city, so the distancing measures will take much work. The minimal activities during the pandemic also meant the reduction of vaccination coverage, so after the pandemic, most of the health officers in Surabaya were fully aware of the resurgence of cases, as happened to many infectious diseases in Indonesia and other countries. For this reason, surveillance and immunization are two key activities that must always be maintained.

As we move beyond the pandemic era, many infectious diseases are resurging, with some regions experiencing incidence rates that exceed pre-pandemic levels [26-28]. In Indonesia, there have already been reports of outbreaks of other vaccine-preventable diseases [29,30], highlighting the need for enhanced surveillance and rapid response measures.

The isolation strategy and several other preventive measures found during the COVID-19 period might not be fully repeated after the pandemic era; however, partial strategies and techniques may be implemented for the prevention efforts of many infectious diseases. The isolation and preventive measurements have already been done, but we may need to strengthen this matter. Immunization is the main factor in protecting people against vaccine-preventable disease outbreaks. All stakeholders in Indonesia should prioritize most of our resources for this purpose. Identifying barriers and developing a better strategy to overcome them may not be waiting for a long time. Public awareness regarding vaccine-preventable diseases, especially diphtheria, must be improved in many ways. Again, some of these issues have already been implemented, but the future needs stronger and better achievements.

This study has several limitations. First, the data were collected cross-sectionally rather than prospectively. The doubtful information could not be clarified by using the surveillance report data cross-sectionally. It would be better if the data were collected prospectively. Second, the microbiological culture was limited to *C. diphtheriae* as per current national policy, though there are ongoing discussions by the Ministry of Health to expand testing to include *C. ulcerans* and *C. pseudotuberculosis* in the future. *C. ulcerans* has already been found in several animals in a few parts of Indonesia.

## Conclusions

The incidence of diphtheria in children during the pandemic was significantly lower compared to the preceding three years. The affected age group, vaccination status, and types of diphtheria remained consistent. Post-pandemic surveillance is crucial for monitoring the disease. Despite the availability of an effective vaccine, diphtheria cases have persisted at high levels for several years, particularly in Indonesia and notably in the East Java province.
